# Temporal dynamics of koala retrovirus plasma RNA load in relation to faecal glucocorticoid metabolites and Chlamydia infection

**DOI:** 10.1099/jgv.0.002147

**Published:** 2025-09-22

**Authors:** Michaela D. J. Blyton, Tamara Keeley, Lewis McKillop, Astrid Van Aggelen, Shali Fischer, Michael Pyne, Keith J. Chappell

**Affiliations:** 1 The University of Queensland, Australian Centre for Ecogenomics, School of Chemistry and Molecular Biosciences, St Lucia, Qld 4072, Australia; 2 Hawkesbury Institute for the Environment, Western Sydney University, New South Wales, 2753, Australia; 3 Currumbin Wildlife Hospital and Foundation, Currumbin, Queensland 4223, Australia; 4 Port Macquarie Koala Hospital, Port Macquarie, New South Wales, 2444, Australia; 5 The University of Queensland, Australian Institute of Bioengineering and Nanotechnology, St Lucia, Queensland, Australia

**Keywords:** Chlamydia, corticosterone, cortisol, disease, koala retrovirus (KoRV), stress

## Abstract

Koala retrovirus (KoRV) is endemic throughout northern koala populations that are currently in steep decline. We have previously found a strong association between KoRV plasma RNA loads and the risk of secondary diseases, including chlamydiosis. However, it is unclear whether (1) KoRV loads are elevated in sick koalas due to the expansion of leucocyte populations; and/or (2) KoRV induces immunosuppression, increasing susceptibility to disease; and/or (3) KoRV and secondary diseases are related through a third variable such as the physiological stress response. Here, we assess the temporal dynamics of KoRV load over a year and, in relation to chlamydia, to explore the causal direction of their relationship. We also investigated co-variation in faecal glucocorticoid metabolites (FGMs: cortisol and corticosterone) with KoRV load and chlamydia. We found that KoRV load was stable within individuals over time. KoRV load did not increase in wild koalas when they began shedding *Chlamydia pecorum* or decrease when they then tested negative, through self-clearance or treatment. Koalas that were treated for chlamydiosis maintained higher KoRV loads than their healthy counterparts. We reveal that higher average KoRV loads are correlated with higher average FGM levels (*R*^2^=0.27), which could indicate that higher KoRV loads lead to higher stress levels or that higher cortisol levels increase KoRV replication through a glucocorticoid response element that we have identified in the KoRV genome. However, this association cannot explain the relationship between average KoRV load and chlamydia because average FGM levels were not significantly higher in koalas that contracted chlamydia or initially higher in those with chlamydial disease. Together, these results provide compelling evidence that KoRV load does not respond to a change in disease status and instead that koalas with consistently high KoRV loads are more likely to develop chlamydiosis, potentially through immunosuppression.

**Impact statement**

Koala retrovirus (KoRV) is ubiquitous in northern koala populations that have suffered severe declines because of multiple threats including disease. We have previously found that the quantity of virus circulating in the koalas’ blood (KoRV load) is associated with chlamydial infection and disease. However, it was not known whether KoRV caused an increased susceptibility to infection, if sick koalas developed higher KoRV load or if their association was the result of another factor such as stress. In this temporal study, we found that KoRV load did not consistently change when koalas became sick or were treated for chlamydia. In fact, KoRV load remained very stable over time and koalas that became infected with chlamydia maintained higher KoRV loads. This suggests that high KoRV load increases a koala’s susceptibility to chlamydia. We also found that there was a significant association between average faecal stress hormone levels and average KoRV load. However, as faecal stress hormones were not associated with chlamydial infection, it appears that stress does not underpin the association between KoRV load and chlamydia. Together, these results indicate that koala retrovirus is an important contributing factor to the chlamydia epidemic threatening this iconic species.

## Data Availability

Amplicon sequencing data for the KoRV *env* hypervariable region used to identify subtype carriage can be accessed via the NCBI SRA database under BioProject PRJNA1262824: accession numbers SAMN48480763 to SAMN48480836. Koala metadata, KoRV load, KoRV subtyping profiles and FGM levels can be obtained from the SEED database: https://www.seed.nsw.gov.au/.

## Introduction

Koalas are now under threat of extinction, with wild populations in the Australian states of New South Wales (NSW) and Queensland (QLD) in steep decline [1]. In these states, koala populations are heavily impacted by disease, particularly *Chlamydia* infections, that threaten population viability through high rates of infertility and mortality [2]. Koala retrovirus (KoRV) is endemic throughout these populations and is strongly associated with secondary diseases [3,6]. KoRV exists as both an endogenous virus (KoRV-A), integrated into the koala germline, and as a wide diversity of exogenous subtypes (KoRV-A-M) that are primarily acquired from the mother at an early age [7,9]. Importantly, these exogenous subtypes appear to produce higher KoRV loads, with the total proportion of KoRV plasma RNA that is from exogenous KoRV positively correlated with total KoRV load [10]. Previous small-scale studies of the association between KoRV load and secondary disease have produced mixed findings. For instance, KoRV RNA was only detected in the blood of a sick koala but not healthy koalas at a Japanese Zoo [11]; increased levels of KoRV plasma RNA were seen in animals with clinical chlamydiosis [12]; Victorian koalas with urogenital tract disease had higher KoRV loads [13]; and in a population of koalas in South Australia, KoRV load was associated with the severity of chlamydial disease [14,15]. However, KoRV load was not associated with the severity of chlamydial disease in two small-scale studies of QLD koalas, and there was no association between KoRV load and urogenital or ocular *Chlamydia pecorum* loads or disease [14,16]; and another study found higher expression of KoRV, primarily attributed to KoRV-D, in healthy koalas than those with chlamydiosis [17]. These contrary findings led to our large-scale study of QLD koalas, which showed that high plasma KoRV loads are strongly associated with chlamydial disease pathologies, including conjunctivitis, cystitis, renal pathology and reproductive pathology, as well as overall poor body condition [3]. Despite these strong associations, a causal relationship has nonetheless not been established and what drives variation in KoRV load is mostly unknown, with exogenous subtype expression only explaining a small proportion of the variation [10]. In this study, we assess the temporal dynamics of KoRV load in relation to chlamydia to explore the causal direction of their relationship. We also investigate one potential underlying driver of variation in KoRV load, the glucocorticoid hormones cortisol, as well as its direct association with chlamydial infection.

There are at least three non-mutually exclusive hypotheses to explain the association between KoRV load and secondary infectious diseases such as chlamydia: (1) KoRV loads are elevated in sick koalas due to the expansion of leucocyte populations; (2) KoRV induces immunosuppression, increasing susceptibility to disease; and (3) KoRV and secondary diseases are related through a third variable. Like other retroviruses, exogenous KoRV is thought to infect the rapidly dividing cells of the immune system [18,20]. In one study, the Peripheral Blood Mononuclear Cell (PBMC) enriched tissue of the spleen showed the highest proviral copy numbers [21]. Further, we have found that buffy coat (white blood cell) samples have higher proviral copy numbers than ear clip tissue samples from the same animals (study available in the online Supplementary Material). Thus, as infections typically induce elevated PBMC counts, chlamydia and other secondary diseases could increase KoRV RNA load in the blood through increased cellular replication of the virus (hypothesis 1). Alternatively, in other retroviruses such as Human immunodeficiency virus (HIV), replication and reintegration in immune cells (PBMCs) typically lead to immunosuppression through immune cell death and/or dysregulation [22,25]. There is some evidence that exogenous KoRV infection may have similar effects on the koala immune system [26,27]. Therefore, KoRV replication may cause immune suppression, leading to an increased risk of secondary diseases (hypothesis 2). However, further mechanistic studies are needed to confirm if and how KoRV causes immune suppression.

**Fig. 1.**
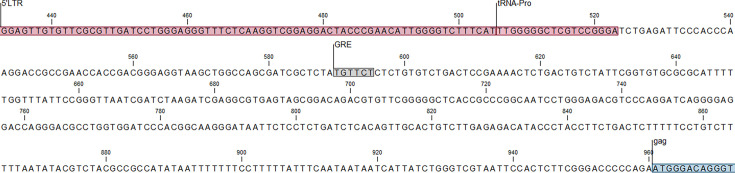
Section of the KoRV-A proviral genome (AF151794; 33) showing the location of the GRE. 5′LTR, tRNA-Pro and *gag* locations were taken from the GenBank annotations.

For the third hypothesis, one potential variable underlying the KoRV load chlamydia association is the physiological stress response. KoRV replication in PBMCs is thought to be suppressed by pre- and post-transcriptional silencing [28] and presumably stimulated by transcription factors. In other retroviruses, the glucocorticoid hormone cortisol can stimulate retrovirus transcription via interaction with a widely conserved glucocorticoid response element (GRE) [29,32]. Examination of the KoRV proviral genome sequence [33] reveals that this GRE is present upstream of the *gag* gene (Fig. 1), raising the distinct possibility that KoRV transcription may be upregulated by physiological stress. Acute stress triggers a complex hormone cascade, initiated by the release of corticotrophin-releasing hormone from the hypothalamus, ultimately culminating in the release of cortisol from the glucocorticoid glands, the major glucocorticoid in mammals, including koalas [34,37]. Although the acute stress response is critical for avoiding predation and danger, chronic stress is known to suppress or compromise the immune system over time, increasing susceptibility to infections [38,40]. Thus, KoRV load may be associated with secondary diseases indirectly through the actions of cortisol as part of the physiological stress response.

Experimental studies of disease are not possible in koalas due to ethical constraints and their status as endangered. Further, mechanistic studies are limited by the availability of appropriate reagents. In lieu of these options, temporal studies can assist in untangling the three hypotheses for KoRV’s association with chlamydia through exploration of if and how KoRV load responds to chlamydial infection. If the association between KoRV load and *Chlamydia* infection is primarily due to elevated PBMC counts driving increased KoRV load (hypothesis 1), then KoRV load should be higher in koalas while they are infected, whereas if KoRV replication is directly responsible for immune suppression (hypothesis 2), then koalas that contract chlamydia should maintain higher KoRV loads than those that remain healthy, but KoRV load should not change when a koala contracts or clears chlamydia. Quigley *et al*. [17] investigated KoRV load over time in 16 koalas of which 2 were treated with antibiotics for chlamydia and 5 became infected with chlamydia at the last time point assessed. This study found that KoRV load was generally stable over time and unaffected by disease status, which is not consistent with the hypothesis that chlamydiosis leads to higher KoRV loads. However, this study had a limited sample size and also did not support the hypothesis that higher KoRV load increases a koala’s susceptibility to disease. In this study, we assessed our three hypotheses by investigating the temporal dynamics of KoRV load using three cohorts of koalas: (1) permanent exhibit koalas at Port Macquarie Koala Hospital that were un-releasable after previous rehabilitation, (2) wild koalas that were tracked for over a year as part of a *Chlamydia* vaccination trial in south-east QLD and (3) wild koalas successfully treated for *Chlamydia* infection at either Port Macquarie Koala Hospital (NSW) or Currumbin Wildlife Hospital (QLD). The exhibit and wild koalas allowed us to assess if and how KoRV load varies over the course of a year in healthy koalas, while the wild and rehabilitation cohorts allowed us to assess if KoRV load increases when a koala contracts *Chlamydia* and/or decreases when they are no longer infected (either through antibiotic treatment or self-clearance).

In addition to KoRV load, we measured faecal cortisol and corticosterone metabolite levels in all three cohorts. These faecal glucocorticoid metabolites were measured in preference to blood hormone levels as they provide an integrated measure of glucocorticoid function and cortisol production over the previous day [41,42]. Whereas, blood cortisol levels are likely to be heavily influenced by the acute stressors of capture and handling, rendering them problematic for the assessment of stress levels in wildlife species [43]. Specifically, we determined if and how these metabolites varied in healthy koalas over time and in response to *Chlamydia* infection. We also measured whether koalas with higher glucocorticoid levels had higher KoRV loads. This enabled us to investigate the role physiological stress may play in KoRV load variation between or within koalas and whether it mediates KoRV’s association with secondary disease.

## Methods

### Koala cohorts

#### Exhibit koalas at Port Macquarie Koala Hospital

Seven exhibit koalas (females=2, males=5) were sampled at Port Macquarie Koala Hospital on two (*n*=5) or three (*n*=2) occasions between May 2023 and February 2024. Samples were collected by veterinary staff during routine health checks. The koalas were wild born and previously brought in for rehabilitation for a range of reasons. However, while these koalas were successfully returned to health, they were deemed non-releasable due to permanent disabilities such as missing claws.

#### Wild koalas involved in *Chlamydia* vaccination study

Between December 2022 and April 2024, blood and faecal samples were collected from 38 wild koalas (30 adults and 8 juveniles) from south-east QLD (28° 08′ 03.8″S, 153° 27′ 03.8″ E) by staff at Currumbin Wildlife Hospital. These koalas were routinely tracked via VHF radio transmitter collars and captured every 3–6 months for health assessment by Currumbin Wildlife Hospital as part of a chlamydia vaccine trial (Queensland University of Technology Animal Ethics Committee Approval 3123). Each koala in the study received two doses of Recombinant MOMP protein (genotypes A, F and G) with Immune Stimulating Complex (ISC) (Zoetis Australia) adjuvant [44]. At each capture, the koalas were tested by Currumbin Wildlife Hospital for the presence of urogenital and ocular *C. pecorum* infection using loop‐mediated isothermal amplification (LAMP) [45]. Most koalas were sampled on two to four occasions, although nine were only sampled on a single occasion and could not be included in the temporal analyses. Of those koalas sampled on multiple occasions, 12 adults (females=9, males=3) and 6 joeys (F=1, M=5) were healthy (not infected with *C. pecorum*) on all sampling occasions, while 10 adults (F=5, M=5) and 1 joey (female) tested positive for Chlamydia at 1 or more sampling time points.

#### Koalas successfully treated for *Chlamydia* infection

Blood and faecal samples were collected from 7 koalas (females=1, males=6) successfully treated for *Chlamydia* infection at Currumbin Wildlife Hospital and 15 (females=5, males=10) from Port Macquarie Koala Hospital. All koalas were first sampled within a day of admission and prior to the administration of antibiotics. Koalas were either treated with doxycycline for typically 2 weeks or chloramphenicol for 2–6 weeks, with the length of treatment determined by the time taken for the koala to first test negative to *C. pecorum*. Between one and three samples were then collected after the completion of antibiotic treatment, the resolution of symptoms and testing negative for ocular and urogenital *C. pecorum*. Where multiple samples were collected after treatment, the first post-treatment samples were used in the before and after comparisons. The majority of koalas were released back to the wild after treatment; however, four koalas from Port Macquarie Koala Hospital were ultimately euthanized after developing secondary conditions after the successful resolution of chlamydia.

### Definition of *Chlamydia* infection

A koala was considered to be ‘infected’ with *C. pecorum* if they returned a quantitative PCR (qPCR)/LAMP-positive ocular or urogenital swab. The infection was considered asymptomatic if the veterinarian did not identify any clinical pathology during physical and ultrasound examination of the koala, whereas the infection was considered symptomatic if the koala had (1) ocular pathology, (2) external urogenital (wet bottom) pathology, (3) renal pathology on ultrasound and/or (4) reproductive pathology on ultrasound that was consistent with the clinical presentation of chlamydia. All koalas treated with antibiotics in this study (all koalas in the chlamydia treatment cohort and three koalas in the *Chlamydia* vaccination trial) were symptomatic, while all other infected koalas were asymptomatic.

### Sample collection

Blood samples were collected to assess KoRV plasma RNA load and KoRV subtypes. Blood was drawn by a qualified veterinarian from the cephalic vein of each koala either under anaesthesia or during conscious restraint. The blood was then transferred to an EDTA-coated tube to prevent clotting and centrifuged at 16,000 ***g*** for 10 min, and the plasma was collected by pipetting, followed by storage at −80 °C.

Faecal samples were collected concurrently with the blood samples to assess faecal cortisol and corticosterone levels. Faecal pellets were either collected from the base of the koala’s enclosure/transport cage or directly from the koala’s cloaca during examination on the same days as the blood samples were drawn. The pellets were placed in zip lock bags and stored at −20 or −80 °C.

### KoRV plasma RNA loads

Total RNA was extracted from the plasma using the High Pure Viral Nucleic Acid Kit (Roche) according to the manufacturer’s instructions with modifications as outlined in Blyton *et al*. [4]. First-strand cDNA was created using Superscript III (Invitrogen) as per Blyton *et al*. [4]. KoRV plasma RNA loads were estimated by qPCR of a 110 bp fragment of the KoRV *pol* gene [11] amplified in triplicate from the plasma cDNA as per Blyton *et al*. [10].

### KoRV subtype profiles

An ~500 bp region of the KoRV *env* gene containing the previously identified hypervariable domain as described in Chappell *et al*. [46] was amplified and sequenced on the Illumina MiSeq. The resulting data was then processed in CLC Genomics Workbench 20 and QIIME 2 [47] according to Blyton *et al*. [4] to determine which subtypes were carried by each koala and in what proportions.

### Faecal cortisol and corticosterone levels

Cortisol and corticosterone metabolites were extracted and analysed as previously reported [48,49]. Briefly, faecal samples were dried overnight at 65 °C, weighed (0.2±0.01 g) and extracted with 5 ml of 80% methanol by overnight agitation. The samples were centrifuged, and the methanol extract was decanted and frozen (−20 °C) until analysis. Faecal extracts were diluted 1 : 10 in assay buffer and analysed for cortisol metabolite levels using a commercially available cortisol enzyme immunoassay (ISWE002; Arbor Assays, USA) and corticosterone using an in-house enzyme immunoassay (CJM006; Coralie Munro, UC Davis, USA) with results evaluated using a BioTek ELx808 plate reader and Gen5 software. The inter- and intra-assay variation for both assays was <14%.

### Statistical analyses

To assess how KoRV load, faecal cortisol and faecal corticosterone varied in healthy koalas over time, linear mixed effects models were fitted using the lme4 package (v: 1.1–35.5) in R [50,51]. Both the exhibit koalas from Port Macquarie Koala Hospital and healthy wild koalas from the vaccine trial were included, with the cohort fitted as a fixed covariate. All samples for each koala were included with koala ID included as a random covariate to account for the repeated measure design. The month, season and whether a sample was collected during the breeding season were all fitted in separate models, with the best model selected by Akaike information criterion [52]. Sex was added as a fixed effect to the best model. Faecal cortisol and faecal corticosterone were square root transformed so that the residuals of the models conformed to the assumption of normality.

The effect of treatment of symptomatic chlamydia on faecal cortisol and faecal corticosterone levels was tested using a linear fixed effects model using the lme4 package (v: 1.1–35.5) in R [50,51]. The institution that the koala was treated at and whether it was a pre-or post-treatment sample were fitted as fixed effects with an interaction between the terms considered. However, as the interaction term was not significant in any of the models, it was removed, and the *P*-values for the primary effects in the reduced model were reported. Koala ID was fitted as a random effect. Faecal cortisol was rank transformed, and faecal corticosterone was square root transformed so that the residuals of the models conformed to the assumption of normality.

To assess if KoRV load and faecal glucocorticoid metabolites covaried within koalas, KoRV load was fitted as the response variable in a linear mixed effects model with koala ID fitted as a random effect and either corticosterone or cortisol fitted as a fixed effect. Whether or not the sample was taken during the breeding season and the state the koala was from were fitted as covariates as our analyses above revealed that these factors had a significant effect on KoRV load. To assess if KoRV load and faecal glucocorticoid metabolite levels were correlated across koalas, the average KoRV load was fitted as the response variable in a linear regression model with state fitted as a covariate. Either average corticosterone or cortisol was fitted as an explanatory variable with the average square root of each metabolite also considered due to the presence of outlying high values. Only samples taken from adult koalas that were healthy at the time of sampling were included in the analysis. This was because KoRV load tended to be more variable and lower in juveniles and the faecal glucocorticoid metabolites were influenced by treatment of symptomatic *Chlamydia* infection.

Significance values were determined using the lmerTest package (3.1–3), and the proportion of variance explained by each variable was determined using the package partR2 [53].

## Results

### Temporal dynamics of KoRV load

#### Little variation in KoRV load over time in healthy koalas

**Fig. 2.**
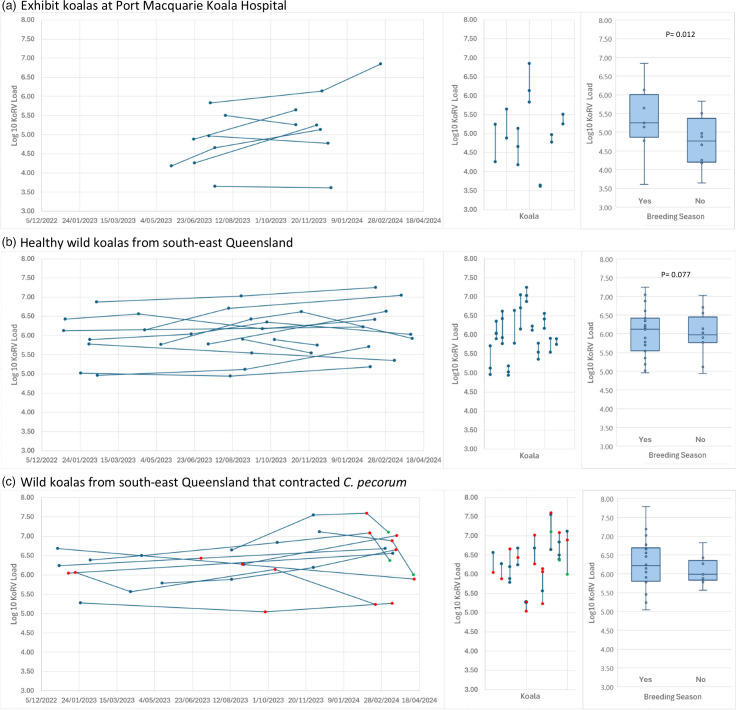
Variation in plasma KoRV RNA load within (**a**) exhibit koalas from Port Macquarie Koala Hospital, (**b**) healthy wild koalas from south-east QLD and (**c**) wild koalas from south-east QLD that contracted *C. pecorum*. Blue points represent samples where the koala was healthy, red points indicated samples from koalas when they tested positive for *C. pecorum* and green points indicated samples post-antibiotic treatment. Lines join samples from the same koala. The left-hand panel shows KoRV load over time, the centre panel shows KoRV load by koala and the right-hand panel shows a box plot of KoRV load by breeding season.

Across the seven exhibit koalas at Port Macquarie Koala Hospital (NSW), the average log10 change in KoRV load per millilitre of plasma between samples was 0.47 (sd=0.31) with an average time between samples of 4 months (123.56 days±sd=38.09; Fig. 2a).

Among the wild koalas tracked as part of the *Chlamydia* vaccine trial in south-east QLD, 12 adults were sampled on 2 or more occasions and were healthy (and not infected with *Chlamydia*) at all those time points. The average log10 change between sampling times was 0.32 (sd=0.21) with an average of 5.3 months (163.5±70.49 days) between samples (Fig. 2b).

Tarlinton *et al*. [12] previously identified that younger koalas generally have lower KoRV loads. Therefore, we examined the KoRV loads of juvenile koalas separately from adults. There were six juvenile koalas that remained healthy (uninfected) throughout the sampling period. Consistent with the findings of Tarlinton *et al*. [12], the juveniles had slightly higher variation in log10 KoRV load between sample points (0.55±0.47) compared with the adults and generally had lower KoRV loads (5.49 vs 6.01; data not shown), although this was not significant (*P*=0.122; Table S1, available in the online Supplementary Material), likely due to small sample size.

Overall, KoRV load remained relatively stable in the healthy koalas over time compared with the observed range between koalas of 3.61 and 7.25 (Fig. 2a, b). Despite this, the adult koalas were found to have significantly higher log10 KoRV loads on average by 0.29 during the breeding season (September to March) compared with the non-breeding season (Tables S2 and S3; linear mixed effects model: *P*=0.004; Fig. 2a, b). However, the breeding season only explained a small proportion of the variation in KoRV load (2.5%), while the majority of the variation was explained by the koala the sample was collected from (Koala ID; 54.1%). The NSW exhibit koalas also had significantly lower log10 KoRV loads by 0.98 on average than the healthy (uninfected) wild QLD koalas (Table S3; *P*=0.007), explaining 30.4% of the variation. No differences in KoRV load were found between the sexes (Table S4, *P*=0.183).

#### No effect of *Chlamydia* infection on KoRV load

Ten of the wild koalas tracked as part of the *Chlamydia* vaccine trial tested positive for *C. pecorum* at one or more sampling time points, with three that were symptomatic subsequently treated at Currumbin Wildlife Hospital with a course of antibiotics and successfully released (Fig. 2c). From this, there were nine transitions from *C. pecorum* negative to positive and three from * C. pecorum* positive to negative (without intervention) for which we had samples. Of the nine transitions from *C. pecorum* negative to positive, KoRV load increased in five and decreased in four, with an average log10 change of 0.03. Similarly, of the three transitions from *C. pecorum* positive to negative, KoRV load increased in two and decreased in four, with an average log10 change of 0.09 (Fig. 2c). Therefore, KoRV load did not appear to change in response to *C. pecorum* infection status. However, it should be noted that most of these cases were asymptomatic. For the three symptomatic koalas, KoRV load decreased in all cases after treatment, which may indicate an effect of treatment (Fig. 2c).

**Fig. 3.**
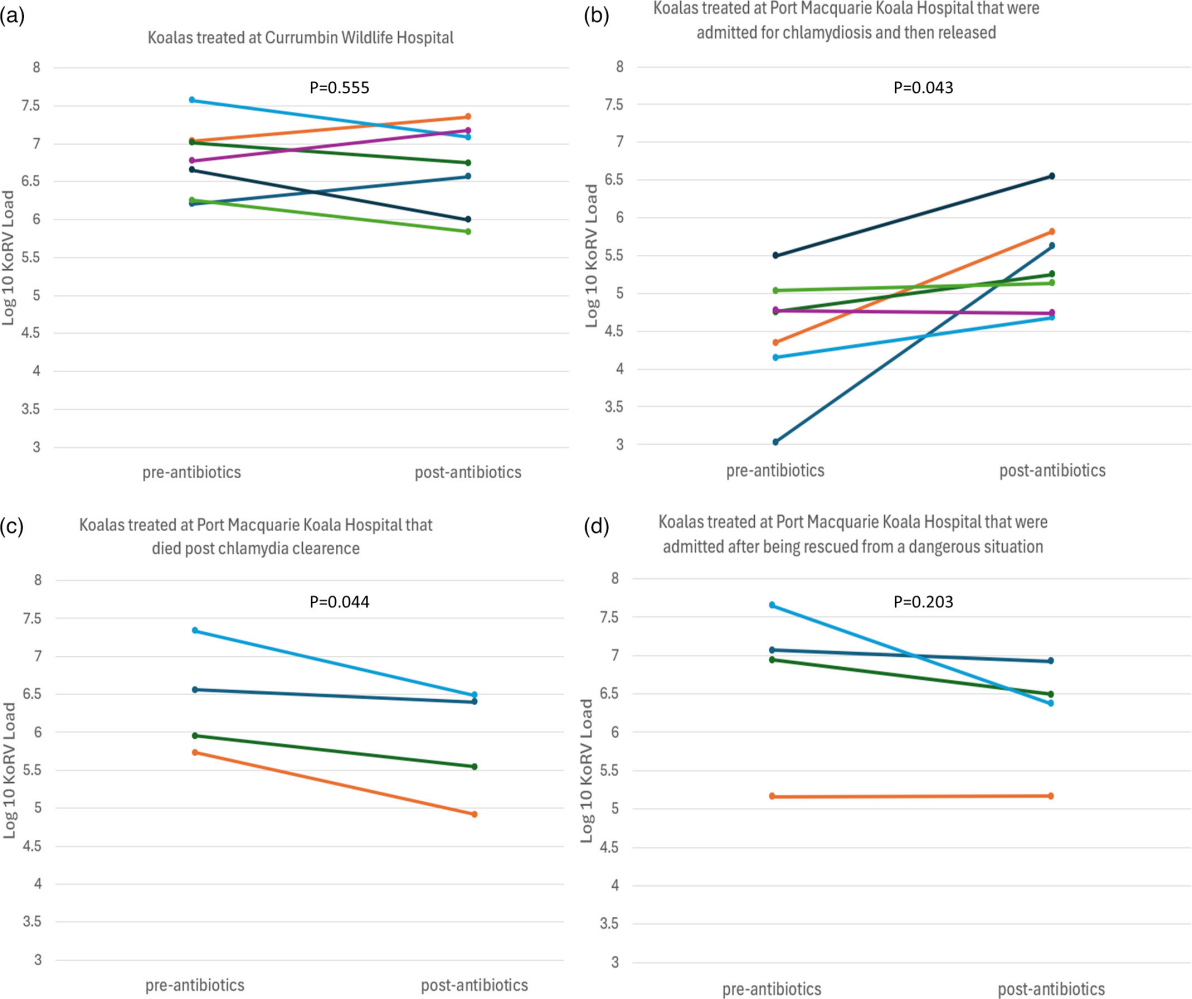
Plasma KoRV RNA load within wild koalas treated for symptomatic *C. pecorum* infection at Currumbin Wildlife Hospital and Port Macquarie Koala Hospital before and after treatment with antibiotics. Lines join samples from the same koala.

To assess if KoRV load was influenced by the resolution of symptomatic chlamydia through antibiotic treatment, we measured KoRV load before and after treatment (between 1 day and 2 weeks after completion of the antibiotic course) for 7 wild koalas brought in for rehabilitation at Currumbin Wildlife Hospital and 15 treated at Port Macquarie Koala Hospital. Overall, there was no significant change in KoRV load with successful chlamydia treatment (paired t-test: *P*=0.738) or for either the Port Macquarie (paired t-test: *P*=0.593) or Currumbin (paired t-test: *P*=0.555) koalas separately (Fig. 3).

However, when examining the responses of the koalas from Port Macquarie Koala Hospital to treatment, we observed an interesting pattern and performed post hoc analyses to support our observations. These tests showed that KoRV load increased in all koalas brought in for chlamydia treatment at Port Macquarie and successfully released (paired t-test: *n*=7; *P*=0.043; Fig. 3b), while it decreased in those koalas that died or were euthanized post-treatment due to other causes such as gut dysbiosis (paired t-test: *n*=4; *P*=0.044; Fig. 3c). Further, KoRV load generally decreased with treatment for koalas brought in for rehabilitation after being rescued from a dangerous situation and were then subsequently found to have symptomatic chlamydia, though this was non-significant (paired t-test: *n*=4; *P*=0.203; Fig. 3d).

#### KoRV load is higher in koalas that contract *Chlamydia* or die during rehabilitation

**Fig. 4.**
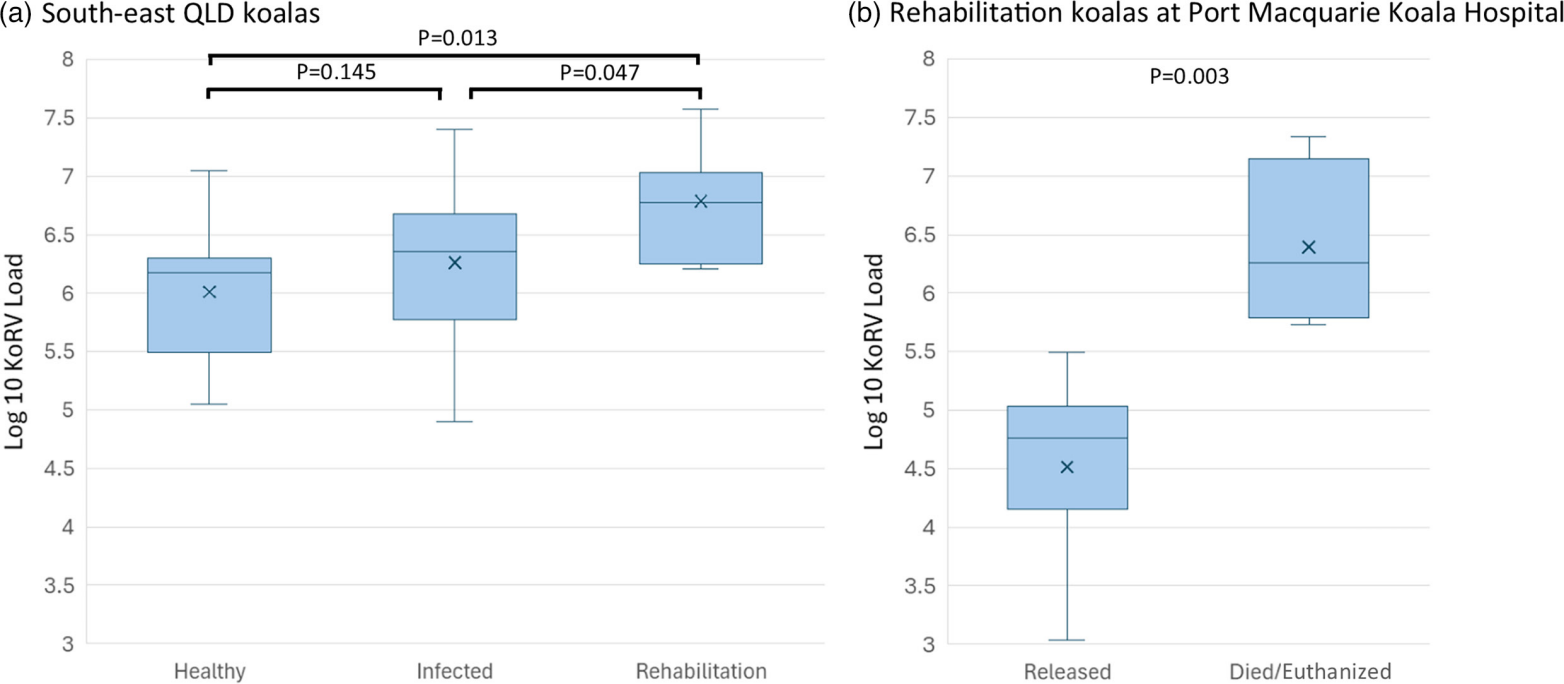
(**a**) Average plasma KoRV RNA load for wild south-east QLD koalas that either remained healthy throughout the study or contracted *C. pecorum* at one or more time points, as well as the initial KoRV RNA loads of koalas admitted to Currumbin Wild Hospital for rehabilitation for chlamydiosis. (**b**) The initial KoRV RNA loads of koalas admitted to Port Macquarie Koala Hospital for chlamydiosis. The X within each box plot indicates the mean value.

KoRV load was found to differ significantly between koalas brought in for rehabilitation for chlamydia, the wild QLD koalas that tested positive for *Chlamydia* on one or more occasions and the wild QLD koalas that remained healthy (ANOVA: *F*=3.44; *P*=0.043). The initial KoRV load of the koalas brought into Currumbin Wildlife Hospital for treatment for chlamydia (mean=6.79±sd=0.48; *n*=7) was significantly higher than the average KoRV load of either the healthy (uninfected) wild QLD koalas tracked as part of the *Chlamydia* vaccine trial (Table S5; *P*=0.013) or those that tested positive for *C. pecorum* on one or more occasions (Table S5; *P*=0.047; Fig. 4a). Among the wild QLD koalas in the vaccine trial, those that tested positive for *C. pecorum* generally had higher average KoRV loads (mean=6.26±sd=0.62; *n*=24) than those koalas that remained healthy (uninfected) throughout the study (6.01±0.60; *n*=9). However, this difference was not significant (one-tailed t-test: *P*=0.145), likely due to the sample size of the healthy cohort and the effect of other factors, such as the koala’s rate of contact with infected koalas.

As we observed a difference between subgroups of koalas at Port Macquarie Koala Hospital in how their KoRV loads responded to chlamydia treatment, we performed post hoc tests to investigate if their initial KoRV loads also differed. Interestingly, those koalas brought in for chlamydia treatment and successfully released had significantly lower initial KoRV loads (4.51±0.78) than those that subsequently died or were euthanized post-treatment (6.40±0.72; two-tailed t-test: *P*=0.003; Fig. 4b). This could not be assessed for koalas from Currumbin Wildlife Hospital as all koalas included in this study from there were successfully released.

### KoRV load is lower in koalas with a higher proportion of subtype KoRV-A

For each koala included in the study, sequencing of the KoRV *env* hypervariable region was performed on a single sample to determine which subtypes of KoRV the koala was carrying and their proportional expression in plasma.

**Fig. 5.**
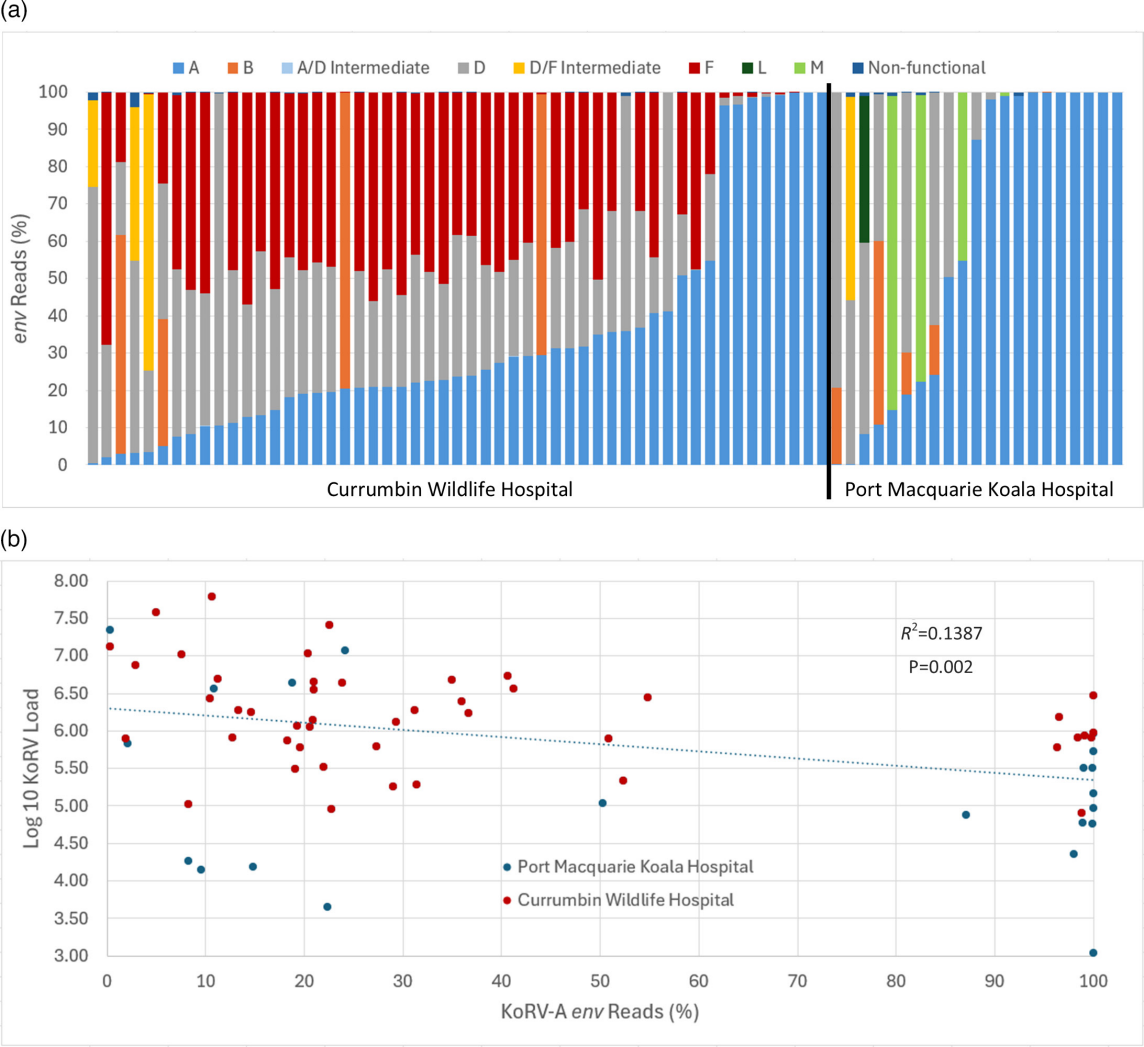
(**a**) Proportion of *env* reads assigned to each KoRV subtype for koalas from Currumbin Wildlife Hospital and Port Macquarie Koala Hospital. (**b**) Correlation between KoRV RNA loads and the proportion of *env* reads that were assigned to KoRV-A.

All 54 koalas from south-east QLD had the endogenous form of KoRV, subtype A, with an average proportion of 35.35%. Only three (5.6%) koalas carried only KoRV-A. The second most prevalent subtype was KoRV-D, which was found in 50 (92.6%) of koalas, followed closely by KoRV-F that was found in 47 (87.0%) koalas. KoRV-B was found in 5 (9.3%) koalas. KoRV-D/F intermediates were found in four koalas, while KoRV-A/D intermediates were found in three. The other KoRV subtypes were not detected, and no new subtypes were identified (Fig. 5a).

The subtype profiles of the 21 koalas from Port Macquarie Koala Hospital differed substantially from those found in south-east QLD. KoRV-A was also detected in all north coast NSW koalas; however, it was found at a higher average proportion (61.3%) compared with south-east QLD (35.4%), and 5 of 21 (23.8%) koalas carried only KoRV-A (Fig. 5a). KoRV-D was found in ten (47.6%) koalas, while KoRV-B was detected in six (28.6%) and KoRV-M was found in four (19.0%). KoRV-A/D and D/F intermediates were each found in one koala. One of the exhibit koalas also carried KoRV-L; however, it should be noted that this koala was not originally from the north coast NSW region but instead came from south of Sydney.

Across all samples, KoRV load was significantly lower in those koalas for which the proportion of KoRV-A in the plasma was higher (linear regression: *R*^2^=0.139, *P*=0.002; Fig. 5b), even after accounting for the koala’s state (Table S6; partial *R*^2^=0.057, *P*=0.026).

### Temporal dynamics of faecal glucocorticoid metabolites

#### Variation in cortisol and corticosterone over time in healthy koalas

There was generally good consensus between the two faecal glucocorticoid metabolites (faecal cortisol and corticosterone *R*^2^=0.757; data not shown).

**Fig. 6.**
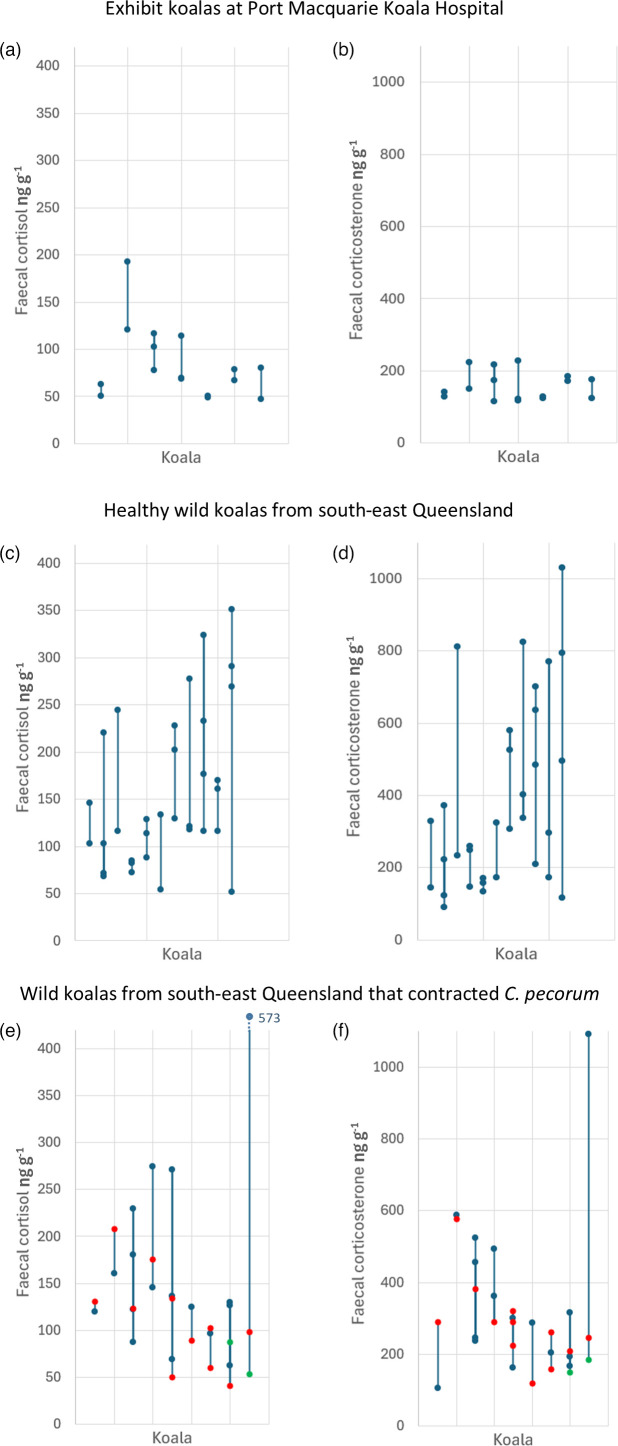
Variation in faecal cortisol (**a, c, e**) and corticosterone (**b, d, f**) within exhibit koalas from Port Macquarie Koala Hospital (**a, b**), healthy wild koalas from south-east QLD (**c, d**) and wild koalas from south-east QLD that contracted *C. pecorum* (**e, f**). Blue points represent samples where the koala was healthy, red points indicated samples from koalas when they tested positive for *C. pecorum* and green points indicated samples post-antibiotic treatment. Lines join samples from the same koala.

For the exhibit koalas at Port Macquarie Koala Hospital (NSW), the average change (irrespective of direction) in faecal cortisol metabolites between consecutive samples from the same koala was 25.37 ng g^−1^ (sd=23.67; Fig. 6a), while faecal corticosterone changed by 45.78 ng g^−1^ (sd=40.33) on average (Fig. 6b). Among the healthy adult wild koalas in south-east QLD, faecal cortisol and corticosterone increased/decreased by 67.77±60.97 and 223.06±181.33 ng g^−1^, respectively, between consecutive samples (Fig. 6c, d). As such, significantly more variation was seen between consecutive samples in the faecal glucocorticoid metabolites for the healthy wild koalas than for the exhibit koalas over a similar period (two-tailed t-test of changes in FGMs between samples: faecal cortisol *P*=0.049; faecal corticosterone *P*=0.006; Fig. 6a–d).

There was no evidence of faecal glucocorticoid metabolite level variation differing between the juvenile and adult healthy wild koalas (Tables S7 and S8; *P*>0.453). The juveniles’ faecal cortisol and corticosterone levels changed by 98.80±82.42 and 136.26±234.75 ng g^−1^, respectively, between consecutive samples (data not shown).

The NSW exhibit koalas had significantly lower faecal cortisol (Table S9; *P*=0.004) and faecal corticosterone (Table S10; *P*=0.002) on average when compared with the healthy wild QLD koalas, explaining 21.4% and 23.4% of the variation in cortisol and corticosterone, respectively. The exhibit koalas’ average faecal cortisol and corticosterone was 84.24 ng g^−1^ (sd=37.94) and 157.64 ng g^−1^ (sd=39.39), while among the healthy adult wild koalas, it was 156.55 ng g^−1^ (sd=80.27) and 384.23 ng g^−1^ (sd=251.32), respectively. Koala ID only explained 14.2% and 12.2% of the variation in faecal cortisol and corticosterone, respectively, indicating that the faecal glucocorticoid metabolites were far more labile than KoRV load within koalas. No seasonal differences (breeding season, season or month) were found in faecal cortisol or faecal corticosterone metabolite levels (Table S11; *P*>0.441; Fig. 6a–d) or between the sexes (Tables S12 and S13; *P*>0.531).

#### Effect of *Chlamydia* infection on cortisol and corticosterone production

There was no evidence that faecal glucocorticoid metabolites change in response to predominantly asymptomatic *Chlamydia* infection. For the wild koalas that tested positive for *C. pecorum*, we had samples for eight transitions from healthy to infected. Cortisol and corticosterone metabolite levels increased in two of these cases and decreased in six, with an average change of −98.30 and −153.86 ng g^−1^, respectively (Fig. 6e, f). Of the three transitions from infected to healthy, cortisol decreased in all three, while corticosterone increased in one instance, with an average change of −15.85 and −56.05 ng g^−1^, respectively. However, there was no overall significant difference in either cortisol and corticosterone metabolite levels between time points where the koala tested positive for *C. pecorum* and those where they tested negative (two-tailed t-test, cortisol: *P*=0.424; corticosterone: *P*=0.608).

**Fig. 7.**
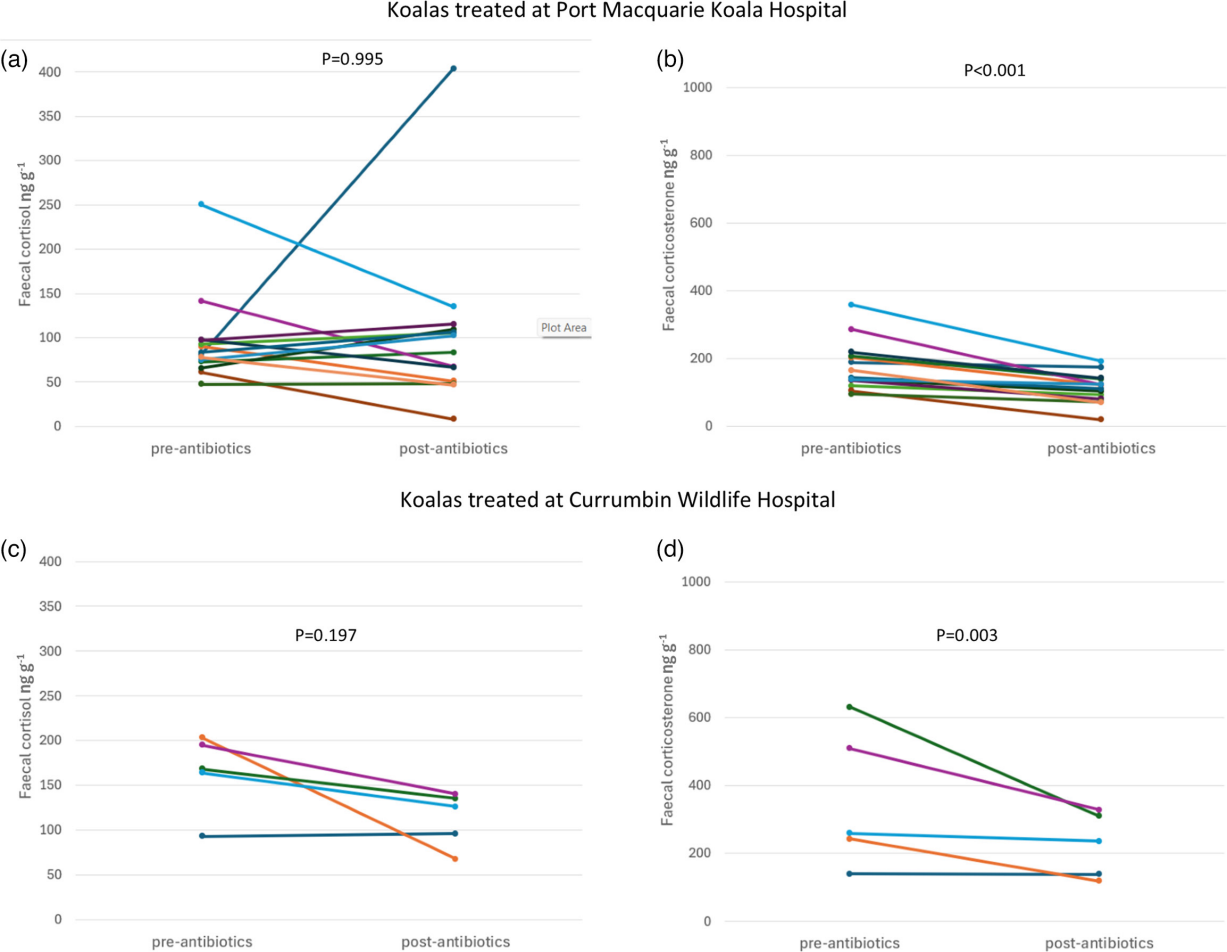
Faecal cortisol (**a, c**) and corticosterone (**b, d**) levels within wild koalas treated for symptomatic *C. pecorum* infection at Port Macquarie Koala Hospital (**a, b**) and Currumbin Wildlife Hospital (**c, d**) before and after treatment with antibiotics. Lines join samples from the same koala.

For the koalas that were treated with antibiotics for symptomatic *Chlamydia* infection, faecal corticosterone metabolite levels significantly decreased between their pre- and post-treatment samples (paired t-test: *P*<0.001). Faecal corticosterone decreased in all 5 koalas treated at Currumbin Wildlife Hospital and all 14 koalas treated at Port Macquarie Koala Hospital by an average of 129.95 and 66.70 ng g^−1^, respectively (Fig. 7). By contrast, the response of faecal cortisol to treatment was less consistent with levels increasing in nine koalas and decreasing in ten, and there was no overall significant change (paired t-test: *P*=0.59; Fig. 7).

#### No difference in cortisol and corticosterone production in koalas that contract chlamydia or die during rehabilitation

There were no significant differences in the initial faecal glucocorticoid metabolite levels of the koalas brought into Currumbin Wildlife Hospital for treatment for chlamydia and the average levels of the healthy or *Chlamydia* positive wild QLD koalas (ANOVA: cortisol *F*=0.84, *P*=0.44, corticosterone *F*=0.14, *P*=0.87).

For the koalas brought in for chlamydia treatment at Port Macquarie Koala Hospital, there was no significant difference in their initial faecal glucocorticoid metabolite levels between koalas that were successfully released compared with those that subsequently died or were euthanized post-treatment (post hoc t-tests: cortisol: *P*=0.282; corticosterone: *P*=0.105; data not shown).

### Relationship between KoRV and faecal glucocorticoid metabolites

**Fig. 8.**
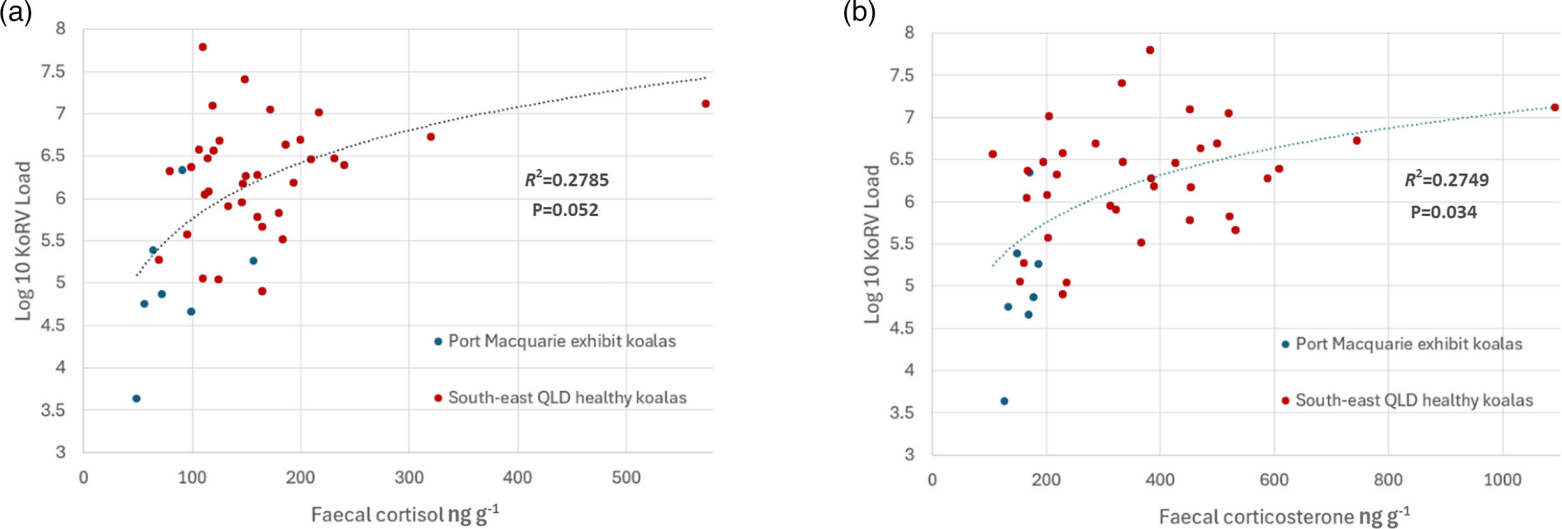
Association between average faecal cortisol (**a**) and corticosterone (**b**) levels and average KoRV plasma RNA load for healthy koalas. Logarithmic trend line shown with associated *R*^2^ value across all samples.

Among samples from adult koalas at time points where they were healthy (exhibit koalas at Port Macquarie Koala Hospital and wild adult QLD koalas at time points where they were uninfected with *C. pecorum*), neither corticosterone nor cortisol was a significant predictor of log10 KoRV load (*P*=0.912 and *P*=0.360) after accounting for koala identity, state and breeding season (Tables S14 and S15; data not shown). However, the average log10 KoRV load was significantly higher in koalas with higher average faecal corticosterone metabolite levels and near significantly higher in koalas with higher average faecal cortisol metabolite levels (Tables S16–S19; corticosterone: *P*=0.040, square root corticosterone: *P*=0.034, cortisol: *P*=0.068, square root cortisol: *P*=0.052) after accounting for state (Fig. 8). Together, these results indicate that within koala variation in KoRV load cannot be explained by changes in faecal glucocorticoid metabolite levels but that those koalas with generally higher faecal glucocorticoid metabolites also had higher average KoRV load.

## Discussion

This study aimed to investigate the temporal dynamics of KoRV load in relation to chlamydia, to provide insights into the direction of causation for the previously reported association between high KoRV load and an increased chance of infection with * C. pecorum* [10,13, 16, 54]. The results of this work have provided compelling evidence that higher KoRV load does indeed increase the risk of *C. pecorum* infection and disease symptoms. In agreement with the findings of Quigley *et al*. [17], we found that KoRV load was very stable within individuals over time, with considerably more variation seen between individuals than within individuals. KoRV load did not increase when wild koalas tested positive for *C. pecorum* or decrease with resolution of infection, either through self-clearance of asymptomatic infection or treatment for symptomatic disease. By contrast, in agreement with our previous findings, koalas that were treated for symptomatic chlamydial disease at Currumbin Wildlife Hospital had higher initial KoRV loads than healthy (uninfected) wild koalas from the same area. There was also a trend for wild koalas that began shedding *C. pecorum* (tested positive) to have higher average KoRV loads. Together, these results show that KoRV load does not respond to a change in infection status and instead that koalas with consistently high KoRV loads are more likely to contract * C. pecorum* and develop chlamydiosis.

Chronically higher glucocorticoid hormone levels are known to cause immune suppression and consequently an increased risk of infection in many species [38,40]. We found that higher average KoRV loads were associated with higher average faecal glucocorticoid metabolite levels. However, this association cannot explain the relationship between average KoRV load and chlamydia because average faecal metabolite levels were not significantly higher in koalas that contracted * C. pecorum* or initially higher in those with symptomatic chlamydial disease. Instead, it is likely that KoRV plasma RNA load is indicative of higher KoRV replication in PBMCs, leading to immune suppression either through cell death or dysregulation. Current understanding of how KoRV activity affects koala immunology is limited, although work has shown that KoRV infection alters the CD4:CD8 gene expression ratio [26]. Reduced CD4:CD8 ratio for other retroviruses is used as a measure of progression of immunosuppression [55]. Further, the envelope protein of KoRV contains a structural element, p15e, which has been shown for other gammaretroviruses, to inhibit lymphocyte activation and alter cytokine expression and thereby contribute to immunosuppression [27,56]. Further, koalas show tissue-specific differences in KoRV, with one study finding the highest proviral copy numbers in the PBMC-enriched tissue of the spleen [21], and we have observed higher proviral copy numbers in buffy coat samples compared with ear clip samples (online supplementary). As with HIV [57], the higher level of KoRV reintegration in PBMCs may disrupt the gene expression by either activating or disrupting transcription of genes that are adjacent to integration sites. Future studies should explicitly focus on elucidating the mechanistic relationship between KoRV load, immune suppression and disease susceptibility to definitively evaluate the causative nature of this relationship.

The growing evidence for the effect of KoRV load on disease risk, combined with the substantive variation in KoRV load between individuals, highlights the need to identify and understand the underlying mechanisms that underpin an individual’s KoRV load. The general temporal stability of KoRV load within individuals over time suggests that animal-level factors, such as genetics, physiology or KoRV subtype profile, are the major determinants of plasma KoRV RNA levels. In this study and in previous work, we have found a negative correlation between KoRV load and the proportion of *env* deep sequencing reads that were endogenous KoRV-A [10]. This is consistent with the partial suppression of endogenous KoRV-A expression, while the exogenous KoRV subtypes obtain high infection levels. The level of expression of the exogenous subtypes is likely to be partly explained by the number and identity of the subtypes carried by an individual [10]. Nonetheless, a 4-log variation in KoRV-A expression is observed between koalas that cannot be explained by subtype profiles. Instead, KoRV-A proviral germline integration sites vary greatly between individual koalas [58], and it is likely that these insertion sites affect the level of KoRV-A transcription.

Despite the general temporal stability of KoRV load within koalas, KoRV load was found to increase slightly during the koala breeding season and vary in response to rehabilitation. The drivers of this within koala variation in KoRV load are currently unknown but may involve modulation of PBMC populations in response to a range of intrinsic and extrinsic factors. Additionally, variation in KoRV subtype proportions over time [17] could explain variation in KoRV load, though the mechanism by which subtype proportions vary is also unknown. We explicitly tested whether variation in glucocorticoid hormone production could explain within koala variation in KoRV load. Indeed, it has been previously found that faecal glucocorticoid metabolite levels are higher in koalas during the breeding season [59]. However, we did not find such a response in this study (potentially due to the limited number of samples per koala across seasons) and within individual variation in KoRV load could not be explained by temporal variation in either faecal corticosterone or cortisol levels.

Our observation that the response of KoRV load to chlamydia treatment varied by whether the koalas at Port Macquarie Koala Hospital were admitted for chlamydial disease or rescued from a dangerous situation is difficult to explain and may simply be a chance occurrence that is not reproducible. Further, the same pattern was not observed at Currumbin Wildlife Hospital where six of the seven koalas were admitted for conjunctivitis, yet KoRV load increased in half and decreased in the other three. However, it is notable that all koalas within the two groups at Port Macquarie responded in the same manner, suggesting that further investigation may be warranted. All koalas received antibiotic treatment; however, some individuals rescued from danger also had injuries that required pain relief and other forms of treatment. It could be speculated that these differences in treatment were responsible for the observed patterns in KoRV load. Alternatively, there may be other aspects of captivity involved, and further research is needed to examine these possibilities.

The other interesting observation from the Port Macquarie rehabilitation cohort was that the initial KoRV load was higher in koalas that died post-treatment compared with those that were successfully released. Further, KoRV load increased in those koalas that were released and decreased in those that died, such that after treatment, KoRV load was comparable between the two groups (Fig. 3b, c). It is possible that koalas with higher KoRV loads are less likely to survive treatment for chlamydia due to KoRV’s putative effects on immune system function or, as KoRV load is strongly associated with body condition [10], it may serve as a general marker of resilience. The contrasting response of KoRV load to chlamydia treatment between the two groups is more puzzling but perhaps is linked to the koalas’ initial KoRV loads, with chlamydia treatment/captivity having a normalizing effect.

We anticipated that there would be an association between faecal cortisol levels and KoRV load due to the predicted interaction of cortisol with a widely conserved GRE within the KoRV 5′ long tandem repeat stimulating retrovirus transcription [29,30]. While within individual variation in KoRV load could not be explained by faecal glucocorticoid metabolite levels, average glucocorticoid metabolite levels and KoRV load were associated. It is conceivable that the lack of relationship within individuals was due to a temporal lag in the effect of cortisol levels on cellular expression of KoRV and circulating plasma RNA load. Conversely, as faecal glucocorticoid metabolites provide an integrated measure of adrenal function and cortisol production over the previous day [60,61], they may not accurately reflect blood cortisol levels and KoRV expression at the time of sampling. An alternative explanation for the association between average faecal glucocorticoid metabolite levels and average KoRV load is that KoRV leads to higher chronic glucocorticoid hormone production through modulation of koala physiology, as occurs, for instance, in cases of HIV-induced cortisol excess [62,63]. Therefore, to determine how they interact, further mechanistic *in vivo* and *in vitro* studies of the association between KoRV and glucocorticoid hormone levels are indicated.

Our assessment of the temporal variation in faecal glucocorticoid metabolite levels revealed strong agreement between cortisol and corticosterone levels in most instances. However, it was notable that while corticosterone decreased in response to treatment for symptomatic *C. pecorum* infection, the response in cortisol was not consistent. The hospital environment likely has a range of acute stressors for koalas, including the stress of handling for health assessment and treatment [64]. It may be that faecal cortisol levels are more affected by these acute stressors, while corticosterone is more indicative of longer-term chronic stress. In any case, it is reassuring that corticosterone levels decreased with treatment, as it is a frequent concern of koala veterinarians that treatment for chlamydia may be counterproductive to the koalas’ overall health due to perceived increases in chronic stress levels associated with their stay in captivity.

In addition to the characterization of temporal patterns in KoRV load and faecal glucocorticoid metabolites, this study has revealed spatial variation between south-east QLD and the north coast of NSW. Both KoRV load and faecal glucocorticoid metabolite levels were lower for koalas in the NSW cohort than the QLD cohort. While this difference could be attributed to the captive vs wild status of the healthy NSW vs QLD koalas, the same patterns in KoRV load and faecal glucocorticoid metabolites were observed among the wild koalas brought in for rehabilitation in both states (Figs4  7). The reason for these regional differences requires further investigation but may be related to environmental factors, the koalas’ genetics and/or KoRV subtype profiles. In a previous study, we identified two new KoRV subtypes, L and M, in NSW that have not been detected in QLD to date [4]. In this study, these subtypes were again detected only in NSW, while KoRV-F was found in the QLD cohort at high prevalence. Further, the average proportion of plasma load that could be attributed to KoRV-A was higher for the NSW cohort than the QLD cohort. With the known association between KoRV subtype profiles and KoRV load [10] and the observed association between average KoRV load and stress levels, these variations in subtype profiles between the regions could play an important role in determining regional differences in koala health and should be considered in conservation management of the species.

## Supplementary material

10.1099/jgv.0.002147 Uncited Table S1.

## Abbreviations

FGM: faecal glucocorticoid metabolite
GRE: glucocorticoid response element
KoRV: koala retrovirus
LAMP: loop‐mediated isothermal amplification
NSW: New South Wales
QLD: Queensland
qPCR: quantitative PCR
