# Supplementary material for: Temporal dynamics of koala retrovirus plasma RNA load in relation to faecal glucocorticoid metabolites and Chlamydia infection
Source: J Gen Virol. 2025 Sep 22;106(9):002147. doi: 10.1099/jgv.0.002147 (PMC12451608; doi:10.1099/jgv.0.002147)
Supplement: Uncited Table S1. [file jgv-106-02147-s001.pdf]

KoRV *pol* proviral copy numbers in PBMCs/buffy coat compared with ear-clip samples

Aim

To assess if KoRV proviral copy numbers are higher in PBMCs compared with ear-clip tissue samples.

Materials and Methods

Koala Capture

Sample collection was carried out under Western Sydney University ethics approval (A15244) and a scientific licence from the NSW Department of Planning, Industry and Environment (SL102726).

A total of 47 koalas were captured and sampled between April and July 2023 from 21 sites on the NSW mid-north coast (northern extent: Coffs Harbour, southern extent: Port Stephens, western extent: Nowendoc). Koalas were located for capture either through diurnal ground searching with the assistance of binoculars or by nocturnal surveys utilizing thermal camera equipped drones. Koalas were captured using the safety line and flagging technique. The captured koalas were then transported from the location of capture a short distance to a field station for health assessment and sample collection. Koalas were anesthetised by the attending veterinarian using a combination of midazolam premedication (benzodiazepine) at 0.3-0.5mg/kg and Alfaxan (Jurox - Alfaxalone 10mg/ml) at 2mg/kg administered intramuscularly into either of gluteal or quadriceps muscle. Complete anaesthesia was then achieved via mask with 3-5% isoflurane at an oxygen flow rate of 2-3 L/min. At completion of the procedure the isoflurane was turned to 0% and the koala maintained on oxygen at a flow rate of 2-3L / min until the koala regained consciousness. When able to support their head, the koala was moved into a recovery basket and monitored visually. After full recovery from anaesthesia koalas were released at the point of capture.

Sample Collection

Blood was drawn by a veterinarian from each koala's cephalic vein under anaesthesia. The blood was then transferred to an EDTA coated tube to prevent clotting and kept on ice in the field. The samples were then centrifuged at 400 g for 10 minutes within 12 hours of collection and the plasma collected by pipetting. For twelve samples the buffy coat (white blood cells) was then skimmed from the surface of the cell pellet using a pipette and frozen in liquid nitrogen in the field before being transferred to a -80 °C freezer for long term storage in the laboratory. In the case of the remaining 35 koalas all but

1 mL of plasma was removed from the cell pellet and the remaining plasma and buffy coat were transferred to a new tube by pipetting. From this, 1 mL of plasma/buffy coat was overlaid onto 0.75 mL of Ficoll® Paque PLUS (Ficoll) (Cytiva, 17144003) and centrifuged at 4 °C and 400 g for 30 minutes with no brake. Isolated PBMCs were then washed twice with 4 °C phosphate buffered saline (PBS) (Merck 6506-OP) before resuspension in 4 mL of freeze down media (heat inactivated foetal bovine serum [Thermofisher, F0926] containing DMSO [Sigma, D1435] [10%, v/v]). Cells were slowly frozen at -1 °C /minute on dry ice before transferring to liquid nitrogen for storage prior to returning from the field and storage at -80 °C.

A 2 mm diameter ear biopsy was taken from the pinna each koala with a single use biopsy punch. The tissue was stored in 100% ethanol at -20 °C.

DNA Extraction

DNA was extracted from the ear-clip samples by Digsol/proteinase K digestion following the method of Bruford *et al.* (1) and eluted in 50 ul of 1x Tris-EDTA. PCR inhibitors were then removed from the extracted DNA using the OneStep PCR inhibitor removal kit (Zymo) following the manufacturer's instructions.

Total genomic DNA was extracted from buffy coat and PBMC samples using the FavorPrep blood genomic DNA mini kit (Favorgen Biotech Corp), as per the manufacturer's instructions.

Quantitative PCR for Copy number determination

A 110 bp fragment of the KoRV *pol* gene and a 123 bp fragment of the koala β-actin gene were amplified from the extracted DNA by quantitative PCR (qPCR) run in triplicate using previously designed primers (2) as per Blyton et al (3). The number of KoRV gene copies per koala cell was estimated for each sample from the number of KoRV *pol* and β-actin molecules per qPCR reaction as follows: $\frac{KoRV\ gene\ copies\ per\ reaction}{(beta{-}actin\ copies\ per\ reaction/14)}$.

Results

The estimated KoRV *pol* copy numbers per cell ranged from 20.49 to 46.74. On average, there were 1.98 more KoRV *pol* copies per PBMC (or buffy coat cell) than per ear tissue cell (paired one tailed t-test: $p = 0.047$; Figure S1). Among the 47 koalas sampled the estimated KoRV *pol* proviral copy numbers were higher in the buffy coat/PBMC samples compared to the ear-clip samples for 30 koalas.

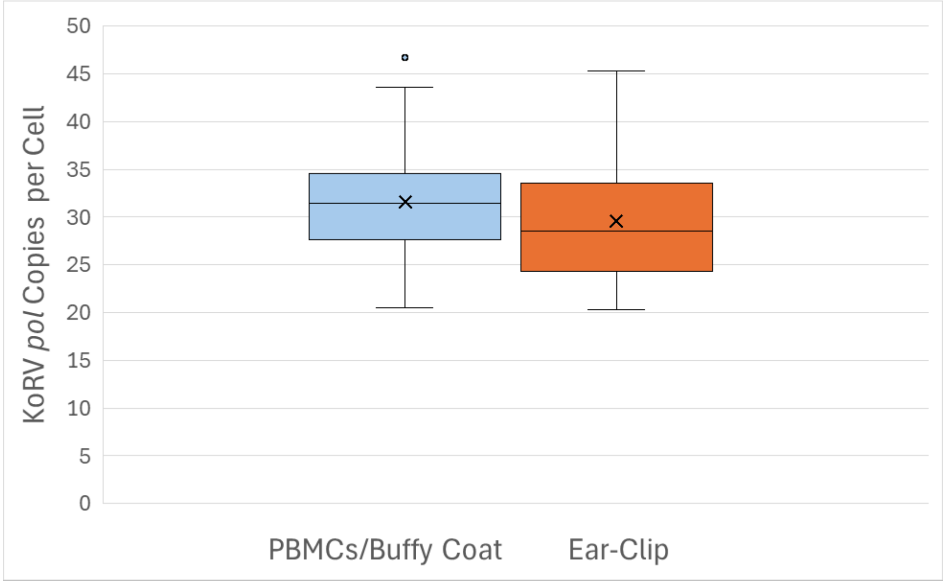

Figure S1: KoRV *pol* proviral copy numbers per cell for PBMC/buffy coat samples compared with ear-clip tissue as estimated by qPCR for 47 wild caught koalas. The X within each box plot indicates the mean value.

Comment

This finding supports the assertion that endogenous KoRV reintegration and/or exogenous KoRV integration occurs more often in PBMCs compared with ear tissue. The average copy number difference between the two cell types was relatively low (approximately 2). However, this is consistent with the high number of endogenous KoRV copies in the koala genome (4) and a comparatively low average level of integration/reintegration, which presumably does not occur in every cell. Further, ear-tissue contains PBMCs and as such the sample types contained overlapping cell sets. While KoRV proviral copy numbers were not higher in the PBMC samples of all koalas compared to ear-clips, this can be attributed to the precision of the qPCR method used to estimate copy numbers, which may not have been sufficient to accurately estimate small copy number differences in individual cases.

References

1. Bruford MW, Hanotte O, Brookfield JKY, Burke T. Multilocus and single-locus DNA fingerprinting. In: Hoelzel AR, editor. Molecular Genetic Analysis of Populations: A Practical Approach. Oxford: IRL Press; 1998. p. 287-336.
2. Kayesh MEH, Yamato O, Rahman MM, Hashem MA, Maetani F, Eiei T, et al. Molecular dynamics of koala retrovirus infection in captive koalas in Japan. Archives of virology. 2019;164(3):757-65.
3. Blyton MDJ, Moore BD, Young P, Chappell K. Geographic patterns of koala retrovirus genetic diversity, endogenization and subtype distributions PNAS. 2022;119(33):e2122680119.
4. Hobbs M, King A, Salinas R, Chen Z, Tsangaras K, Greenwood AD, et al. Long-read genome sequence assembly provides insight into ongoing retroviral invasion of the koala germline. Scientific reports. 2017;7(1):1-9.

Table S1: Linear mixed model fit by REML of difference in KoRV load between adult and juvenile koalas. t-tests use Satterthwaite's method. 47 observations from 18 koalas (6 juvenile and 12 healthy adults from the wild QLD cohort).

Random Effects					
**Group Name**	**Variance**	**Std.Dev.**			
koala.ID	0.2797	0.5289			
Residual	0.1750	0.4183			
Fixed Effects					
**Variable**	**Estimate**	**Std. Error**	**Df**	**t-value**	**Pr(>|t|)**
Intercept	5.9969	0.1727	16.5937	34.732	<2e-16
Cohort: Juvenile	-0.4851	0.2973	16.3634	-1.632	0.122

Table S2: AIC values for linear mixed effects models for KoRV load of breeding season, season and month with cohort (PMKH exhibit or healthy wild QLD koalas) and koala ID fitted as co-variates

**Model including**	**AIC**
Breeding Season	84.31272
Season	92.86391
Month	100.2186

Table S3: Linear mixed model fit by REML of KoRV load by breeding season and cohort. 51 observations from 19 koalas (7 PMKH exhibit koalas and 12 healthy adults from the wild QLD cohort) t-tests use Satterthwaite's method

Random Effects					
**Group Name**	**Variance**	**Std.Dev.**			
koala.ID	0.40855	0.6392			
Residual	0.09478	0.3079			
Fixed Effects					
**Variable**	**Estimate**	**Std. Error**	**Df**	**t-value**	**Pr(>|t|)**
Intercept	5.81254	0.20264	20.64559	28.684	< 2e-16
Cohort: PMKH.Exhibit	-0.98076	0.31887	17.35818	-3.076	0.00673
Breeding Season: Yes	0.29483	0.09555	31.91869	3.086	0.00418

Table S4: Linear mixed model fit by REML of KoRV load by breeding season, cohort and sex. 51 observations from 19 koalas (7 PMKH exhibit koalas and 12 healthy adults from the wild QLD cohort) t-tests use Satterthwaite's method

Random Effects					
**Group Name**	**Variance**	**Std.Dev.**			
koala.ID	0.38349	0.6193			
Residual	0.09498	0.3082			
Fixed Effects					
**Variable**	**Estimate**	**Std. Error**	**Df**	**t-value**	**Pr(>|t|)**
Intercept	5.68982	0.21630	19.40456	26.305	< 2e-16
Cohort: PMKH.Exhibit	-1.19826	0.34703	16.09470	-3.453	0.00325
Breeding Season: Yes	0.29988	0.09569	31.73436	3.134	0.00370
Sex: Male	0.47117	0.33817	15.92779	1.393	0.18267

Table S5: Pairwise comparisons (post-hoc t-tests) of KoRV load between koalas that received rehabilitation for Chlamydia at Currumbin Wildlife Hospital (n =7), wild QLD koalas that tested positive for Chlamydia (n = 24) and those that remained healthy/uninfected (n = 9). Note that three koalas that remained healthy during the sampling window tested positive for Chlamydia outside the sampling window and so were included in the Chlamydia positive group for this analysis. Eleven koalas that were only sampled on one occasion for KoRV load and thus not included in the temporal analyses were included in the Chlamydia positive group for this analysis.

**Comparison**	**Estimate**	**Std. Error**	**t-value**	**Pr(>|t|)**
Intercept with the Chlamydia positive group as the reference	6.2619	0.1216	51.476	<2e-16
Chlamydia negative vs positive	-0.2501	0.2329	-1.074	0.2899
Rehabilitation vs Chlamydia positive	0.5252	0.2560	2.052	0.0473
Rehabilitation vs Chlamydia negative	0.7753	0.3003	2.582	0.0139

Table S6: Linear regression of KoRV load by the proportion of plasma RNA that was KoRV-A with State included as a covariate. 67 koalas were included in the analysis (21 from NSW including both exhibit and rehabilitation koalas and 46 from Queensland including rehabilitation and wild)

**Variable**	**Estimate**	**Std. Error**	**t-value**	**Pr(>|t|)**
Intercept	5.580395	0.240318	23.221	< 2e-16
Proportion of RNA that was KoRV-A	-0.006344	0.002786	-2.277	0.026119
State: QLD	0.842321	0.220474	3.820	0.000304

Table S7: Linear mixed model fit by REML of difference in square root transformed faecal cortisol between adult and juvenile koalas. t-tests use Satterthwaite's method. 49 observations from 18 koalas (6 juvenile and 12 healthy adults from the wild QLD cohort).

Random Effects					
**Group Name**	**Variance**	**Std.Dev.**			
koala.ID	1.096	1.047			
Residual	10.032	3.167			
Fixed Effects					
**Variable**	**Estimate**	**Std. Error**	**Df**	**t-value**	**Pr(>|t|)**
Intercept	12.0902	0.6276	15.3452	19.266	3.62e-12
Cohort: Juvenile	0.8668	1.1289	16.5124	0.768	0.453

Table S8: Linear mixed model fit by REML of difference in square root transformed faecal corticosterone between adult and juvenile koalas. t-tests use Satterthwaite's method. 49 observations from 18 koalas (6 juvenile and 12 healthy adults from the wild QLD cohort).

Random Effects					
**Group Name**	**Variance**	**Std.Dev.**			
koala.ID	10.24	3.201			
Residual	31.08	5.575			
Fixed Effects					
**Variable**	**Estimate**	**Std. Error**	**Df**	**t-value**	**Pr(>|t|)**
Intercept	18.5708	1.3487	14.4922	13.770	1.01e-09
Cohort: Juvenile	0.9526	2.4070	15.7206	0.396	0.698

Table S9: Linear mixed model fit by REML of square root transformed faecal cortisol by cohort. 50 observations from 19 koalas (7 PMKH exhibit koalas and 12 healthy adults from the wild QLD cohort) t-tests use Satterthwaite's method

<table>
<tr><td colspan="6">Random Effects</td></tr>
<tr><th>Group Name</th><th>Variance</th><th>Std.Dev.</th><td colspan="3" rowspan="3"></td></tr>
<tr><td>koala.ID</td><td>1.412</td><td>1.188</td></tr>
<tr><td>Residual</td><td>6.432</td><td>2.536</td></tr>
<tr><td colspan="6">Fixed Effects</td></tr>
<tr><th>Variable</th><th>Estimate</th><th>Std. Error</th><th>Df</th><th>t-value</th><th>Pr(>|t|)</th></tr>
<tr><td>Intercept</td><td>12.0678</td><td>0.5612</td><td>16.6056</td><td>21.502</td><td>1.48e-13</td></tr>
<tr><td>Cohort: PMKH.Exhibit</td><td>-3.1013</td><td>0.9611</td><td>19.9286</td><td>-3.227</td><td>0.00424</td></tr>
</table>

Table S10: Linear mixed model fit by REML of square root transformed faecal corticosterone by cohort. 50 observations from 19 koalas (7 PMKH exhibit koalas and 12 healthy adults from the wild QLD cohort) t-tests use Satterthwaite's method

<table>
<tr><td colspan="6">Random Effects</td></tr>
<tr><th>Group Name</th><th>Variance</th><th>Std.Dev.</th><td colspan="3" rowspan="3"></td></tr>
<tr><td>koala.ID</td><td>4.336</td><td>2.082</td></tr>
<tr><td>Residual</td><td>22.883</td><td>4.784</td></tr>
<tr><td colspan="6">Fixed Effects</td></tr>
<tr><th>Variable</th><th>Estimate</th><th>Std. Error</th><th>Df</th><th>t-value</th><th>Pr(>|t|)</th></tr>
<tr><td>Intercept</td><td>18.582</td><td>1.030</td><td>18.012</td><td>18.046</td><td>5.57e-13</td></tr>
<tr><td>Cohort: PMKH.Exhibit</td><td>-6.121</td><td>1.768</td><td>21.657</td><td>-3.462</td><td>0.00226</td></tr>
</table>

Table S11: AIC and p-values for linear mixed effects models for square root transformed faecal cortisol and corticosterone of breeding season, season and month with cohort (PMKH exhibit or healthy wild QLD koalas) and koala ID fitted as co-variates

Model including	Cortisol AIC	Cortisol p-value	Corticosterone AIC	Corticosterone p-value
Breeding Season	247.593	0.432271	306.9858	0.89823
Season	247.474	0.925058	303.8337	0.939750
Month	234.0596	0.9956	276.7453	0.7883

Table S12: Linear mixed model fit by REML of square root transformed faecal cortisol by cohort and sex. 50 observations from 19 koalas (7 PMKH exhibit koalas and 12 healthy adults from the wild QLD cohort) t-tests use Satterthwaite's method

<table>
<tr><td colspan="6">Random Effects</td></tr>
<tr><th>Group Name</th><th>Variance</th><th>Std.Dev.</th><td colspan="3" rowspan="3"></td></tr>
<tr><td>koala.ID</td><td>1.626</td><td>1.275</td></tr>
<tr><td>Residual</td><td>6.389</td><td>2.528</td></tr>
<tr><td colspan="6">Fixed Effects</td></tr>
<tr><th>Variable</th><th>Estimate</th><th>Std. Error</th><th>Df</th><th>t-value</th><th>Pr(>|t|)</th></tr>
<tr><td>Intercept</td><td>11.8749</td><td>0.6468</td><td>17.4244</td><td>18.360</td><td>7.67e-13</td></tr>
<tr><td>Cohort: PMKH.Exhibit</td><td>-3.4040</td><td>1.0940</td><td>18.6757</td><td>-3.111</td><td>0.00583</td></tr>
<tr><td>Sex: Male</td><td>0.6702</td><td>1.0471</td><td>17.0957</td><td>0.640</td><td>0.53062</td></tr>
</table>

Table S13: Linear mixed model fit by REML of square root transformed faecal corticosterone by cohort and sex. 50 observations from 19 koalas (7 PMKH exhibit koalas and 12 healthy adults from the wild QLD cohort) t-tests use Satterthwaite's method

<table>
<tr><td colspan="6">Random Effects</td></tr>
<tr><th>Group Name</th><th>Variance</th><th>Std.Dev.</th><td colspan="3" rowspan="3"></td></tr>
<tr><td>koala.ID</td><td>5.032</td><td>2.243</td></tr>
<tr><td>Residual</td><td>22.930</td><td>4.789</td></tr>
<tr><td colspan="6">Fixed Effects</td></tr>
<tr><th>Variable</th><th>Estimate</th><th>Std. Error</th><th>Df</th><th>t-value</th><th>Pr(>|t|)</th></tr>
<tr><td>Intercept</td><td>18.4257</td><td>1.1902</td><td>18.4408</td><td>15.481</td><td>5.14e-12</td></tr>
<tr><td>Cohort: PMKH.Exhibit</td><td>-6.3658</td><td>2.0155</td><td>19.8733</td><td>-3.158</td><td>0.00497</td></tr>
<tr><td>Sex: Male</td><td>0.5441</td><td>1.9264</td><td>18.1375</td><td>0.282</td><td>0.78079</td></tr>
</table>

Table S14: Linear mixed model fit by REML of KoRV load by square root transformed cortisol, breeding season, and state. 79 observations from 42 koalas (7 PMKH exhibit koalas, 14 healthy adults and 21 koalas that tested positive for Chlamydia from the wild QLD cohort) t-tests use Satterthwaite's method. Note only samples from timepoints where the koalas were healthy were included in the model.

Random Effects					
**Group Name**	**Variance**	**Std.Dev.**			
koala.ID	0.42772	0.6540			
Residual	0.07301	0.2702			
Fixed Effects					
**Variable**	**Estimate**	**Std. Error**	**Df**	**t-value**	**Pr(>|t|)**
Intercept	4.98322	0.29814	56.53602	16.714	< 2e-16
SQRT Cortisol	-0.01507	0.01629	46.41482	-0.925	0.35966
State: QLD	1.28008	0.28832	39.24367	4.440	7.13e-05
Breeding Season: Yes	0.28294	0.07456	37.75932	3.795	0.00052

Table S15: Linear mixed model fit by REML of KoRV load by square root transformed corticosterone, breeding season, and state. 79 observations from 42 koalas (7 PMKH exhibit koalas, 14 healthy adults and 21 koalas that tested positive for Chlamydia from the wild QLD cohort) t-tests use Satterthwaite's method. Note only samples from timepoints where the koalas were healthy were included in the model.

Random Effects					
**Group Name**	**Variance**	**Std.Dev.**			
koala.ID	0.41022	0.6405			
Residual	0.07727	0.2780			
Fixed Effects					
**Variable**	**Estimate**	**Std. Error**	**Df**	**t-value**	**Pr(>|t|)**
Intercept	4.860043	0.281455	51.138091	17.268	< 2e-16
SQRT Corticosterone	-0.00105	0.009407	45.653584	-0.111	0.911982
State: QLD	1.233629	0.283503	39.201807	4.351	9.37e-05
Breeding Season: Yes	0.284835	0.076518	38.132379	3.722	0.000635

Table S16: Linear regression model of average KoRV load by faecal corticosterone and state. 42 koalas (7 PMKH exhibit koalas, 14 healthy adults and 21 koalas that tested positive for Chlamydia from the wild QLD cohort) Note only samples from timepoints where the koalas were healthy were included in the calculation of average values.

Variable	Estimate	Std. Error	t-value	Pr(>|t|)
Intercept	4.7893183	0.2678952	17.878	< 2e-16
Corticosterone	0.0012199	0.0005744	2.124	0.04009
State: QLD	1.0228403	0.3011982	3.396	0.00159

Table S17: Linear regression model of average KoRV load by square root transformed faecal corticosterone and state. 42 koalas (7 PMKH exhibit koalas, 14 healthy adults and 21 koalas that tested positive for Chlamydia from the wild QLD cohort) Note only samples from timepoints where the koalas were healthy were included in the calculation of average values.

Variable	Estimate	Std. Error	t-value	Pr(>|t|)
Intercept	4.33649	0.38706	11.204	9.3e-14
SQRT Corticosterone	0.05138	0.02340	2.196	0.03411
State: QLD	0.96958	0.30903	3.138	0.00324

Table S18: Linear regression model of average KoRV load by faecal cortisol and state. 42 koalas (7 PMKH exhibit koalas, 14 healthy adults and 21 koalas that tested positive for Chlamydia from the wild QLD cohort) Note only samples from timepoints where the koalas were healthy were included in the calculation of average values.

Variable	Estimate	Std. Error	t-value	Pr(>|t|)
Intercept	4.778311	0.277177	17.239	< 2e-16
Cortisol	0.002439	0.001299	1.878	0.067938
State: QLD	1.082228	0.298186	3.629	0.000815

Table S19: Linear regression model of average KoRV load by square root transformed faecal cortisol and state. 42 koalas (7 PMKH exhibit koalas, 14 healthy adults and 21 koalas that tested positive for Chlamydia from the wild QLD cohort) Note only samples from timepoints where the koalas were healthy were included in the calculation of average values.

Variable	Estimate	Std. Error	t-value	Pr(>|t|)
Intercept	4.27541	0.43505	9.828	4.18e-12
SQRT Cortisol	0.07857	0.03924	2.002	0.05223
State: QLD	1.00190	0.31021	3.230	0.00252
